# Quantitative trait locus mapping of genes that control body length and plasma insulin-like growth factor 1 level in mice

**DOI:** 10.1186/1756-0500-5-547

**Published:** 2012-10-02

**Authors:** Jun-ichi Suto

**Affiliations:** 1 Agrogenomics Research Center, National Institute of Agrobiological Sciences, Tsukuba, Ibaraki, 305-8634, Japan

**Keywords:** *A*^*y*^ allele, Body length, Plasma IGF1 levels, Quantitative trait locus (QTL)

## Abstract

**Background:**

The *A*^*y*^ allele at the agouti locus causes obesity and promotes linear growth in mice. The effect of the *A*^*y*^ allele on obesity has been extensively investigated, whereas its effect on body length is only poorly analyzed. To gain insight into the genetic control of body length, quantitative trait locus (QTL) analysis was performed in F_2_ female mice produced by crossing C57BL/6 J females and DDD.Cg-*A*^*y*^ males. A congenic DDD.Cg-*A*^*y*^ strain was established by introgressing the *A*^*y*^ allele from the B6.Cg-*A*^*y*^ strain by backcrossing for 12 generations. DDD.Cg-*A*^*y*^ females were longer than B6.Cg-*A*^*y*^ females; therefore, QTLs that interact with the *A*^*y*^ allele may be identified for body length. In addition, QTL analysis was also performed for plasma insulin-like growth factor 1 (IGF1) levels because IGF1 is known to play essential roles in growth and development. If QTLs for IGF1 levels coincide with those for body length, we can gain endocrinological insight into the QTLs for body length.

**Results:**

Correlations between body length and IGF1 levels were statistically significant in F_2_ populations. For body length, two significant QTLs were identified on chromosomes 15 and 17. For IGF1 levels, three significant QTLs were identified on chromosomes 10, 12, and 19. QTLs on chromosomes 12 and 19 appeared to be novel, and the latter interacted with the *A*^*y*^ allele.

**Conclusion:**

QTLs for body length and IGF1 levels contained candidate genes that were components of the growth hormone/insulin-like growth factor axis. However, there was no overlap between QTLs for these two traits. Contrary to our expectations, QTLs that interacted with the *A*^*y*^ allele were identified not for body length but for IGF1 levels. Body length and IGF1 levels were, thus, controlled by different sets of genes.

## Background

Traditionally, five single gene obesity mutations, *Cpe*^*fat*^, *Tub*^*tub*^, *Lep*^*ob*^, *Lepr*^*db*^, and *A*^*y*^, have been identified in mice [1]. Among the five mutations, only the *A*^*y*^ allele is dominant and homozygous lethal; therefore, living *A*^*y*^ mice are invariably heterozygotes. Obesity in *A*^*y*^ mice is moderate and occurs late compared with that in the other four mutants. The *A*^*y*^ allele is known not only to cause obesity but also to promote linear growth [2].

In normal mice, the agouti gene is expressed only in the skin [3,4], and it regulates pigmentation by serving as an inverse agonist of the melanocortin 1 receptor [5,6]. However, in *A*^*y*^ mice, the *A*^*y*^ allele is associated with a large deletion, causing agouti gene expression to be aberrantly controlled by the unrelated *Raly* gene promoter and leading to its ectopic overexpression [4,7-9]. As a result, *A*^*y*^ mice have a yellow coat color and develop maturity onset obesity. Obesity in *A*^*y*^ mice is believed to be a consequence of the agouti protein serving as a constitutive antagonist of the melanocortin 3 receptor (MC3R) and melanocortin 4 receptor (MC4R) by mimicking the action of the agouti-related protein [10-12].

Two mouse strains congenic for the *A*^*y*^ allele are available to date: B6.Cg-*A*^*y*^ (C57BL/6 J background, hereafter B6-*A*^*y*^) and KK.Cg-*A*^*y*^ (KK/Ta background, hereafter KK-*A*^*y*^) strains. We developed a novel strain congenic for the *A*^*y*^ allele in an inbred DDD/Sgn (hereafter DDD) strain background, i.e., DDD.Cg-*A*^*y*^ (hereafter DDD-*A*^*y*^) strain [13]. DDD-*A*^*y*^ females are characterized by their massive obesity compared with KK-*A*^*y*^ and B6-*A*^*y*^ females [14], i.e., the average body weight at 16 weeks was 54.2 g in DDD-*A*^*y*^, 52.2 g in KK-*A*^*y*^, and 38.5 g in B6-*A*^*y*^. Although KK-*A*^*y*^ and B6-*A*^*y*^ females did not weigh more than 60 g (body weights were measured by 29 weeks), DDD-*A*^*y*^ females weighed more than 60 g at 19 weeks and older and some weighed more than 70 g by 22 weeks. The magnitude of phenotypic effect of the *A*^*y*^ allele was thus strongly influenced by the genetic background.

To determine the genetic basis of obesity in DDD-*A*^*y*^ mice and to determine whether or not their high body weight was because of the presence of DDD background-specific modifiers, quantitative trait locus (QTL) analyses for body weight and obesity (defined by body mass index, BMI) were previously performed in two types of F_2_ female mice [F_2_*A*^*y*^ (F_2_ mice with the *A*^*y*^ allele) and F_2_ non- *A*^*y*^ mice (F_2_ mice without the *A*^*y*^ allele)] produced by crossing C57BL/6 J females and DDD-*A*^*y*^ males [14]. The presence of DDD background-specific modifiers was not confirmed, and a multifactorial basis for obesity in DDD-*A*^*y*^ females was revealed.

In this study, the genetic basis of body length was analyzed in the same F_2_ population. In addition to the results of the analysis of body weight, we will gain insight into the genetic control of body size because body length also serves as a representative body size parameter. Furthermore, the *A*^*y*^ allele is known not only to cause obesity but also to promote linear growth [2]. The effect of the *A*^*y*^ allele on body weight has been extensively investigated, whereas its effect on body length is only poorly analyzed [15,16]. As with obesity, the effect of the *A*^*y*^ allele on body length was considered to be mediated by the melanocortin 4 receptor [11]. DDD.Cg-*A*^*y*^ strain was longer than B6.Cg-*A*^*y*^ strain; therefore, DDD background-specific modifiers may be identified for body length.

In addition, QTL analysis was also performed for plasma insulin-like growth factor 1 (IGF1) levels because QTLs identified for body length contained candidate genes that were components of the growth hormone/insulin-like growth factor axis and because IGF1 is known to play essential roles in growth and development [17-21]. Thus, we hypothesized that some QTLs for IGF1 levels will be involved in the control of body length. If QTLs for IGF1 levels coincide with those for body length, we can gain endocrinological insight into the QTLs for body length.

## Results

Body length and plasma IGF1 levels in parental, F_1_, and F_2_ female mice are summarized in Table 1. In parental female mice, *A*^*y*^ mice were significantly longer and had significantly higher IGF1 levels than non-*A*^*y*^ mice. Furthermore, DDD-*A*^*y*^ females were significantly longer and had significantly higher IGF1 levels than B6-*A*^*y*^ females and DDD females were significantly longer and had significantly higher IGF1 levels than B6 females. In F_2_ females, *A*^*y*^ mice were significantly longer than non-*A*^*y*^ mice, but IGF1 levels did not significantly differ between *A*^*y*^ and non-*A*^*y*^ mice.

**Table 1:** **Mean ± S.E. body length and plasma IGF1 levels in parental, F**_**1**_**, and F**_**2 **_**female mice**

**Mice**	**n**	**Body length (mm)**	**IGF1 (ng/ml)**
DDD-*A*^*y*^	8	102.39 ± 0.43 ^c, d^	679.3 ± 18.9 ^d, h^
DDD	9	98.22 ± 0.37 ^e^	603.7 ± 18.8 ^e^
B6-*A*^*y*^	5	96.79 ± 0.71 ^e^	465.2 ± 27.8 ^i^
B6	7	90.53 ± 0.55	346.6 ± 12.7
F_1_ non-*A*^*y*^	7	nd ^f^	426.9 ± 21.2
F_1 _*A*^*y*^	7	nd ^f^	443.4 ± 14.3
F_2_ non-*A*^*y*^	148 (137) ^a^	95.42 ± 0.27 ^g^	459.5 ± 5.8
F_2 _*A*^*y*^	150 (139) ^b^	99.73 ± 0.22	445.6 ± 5.8

^a^ The number of F_2_ mice is 148 for plasma IGF1 levels and 137 for body length.

^b^ The number of F_2_ mice is 150 for plasma IGF1 levels and 139 for body length.

^c^ Significant difference (P < 0.0001) versus DDD.

^d^ Significant difference (P < 0.0001) versus B6-*A*^*y*^.

^e^ Significant difference (P < 0.0001) versus B6.

^f^ nd, not determined.

^g^ Significant difference (P < 0.0001) versus F_2_*A*^*y*^.

^h^ Significant difference (P < 0.05) versus DDD.

^i^ Significant difference (P < 0.01) versus B6.

**Figure 1:**
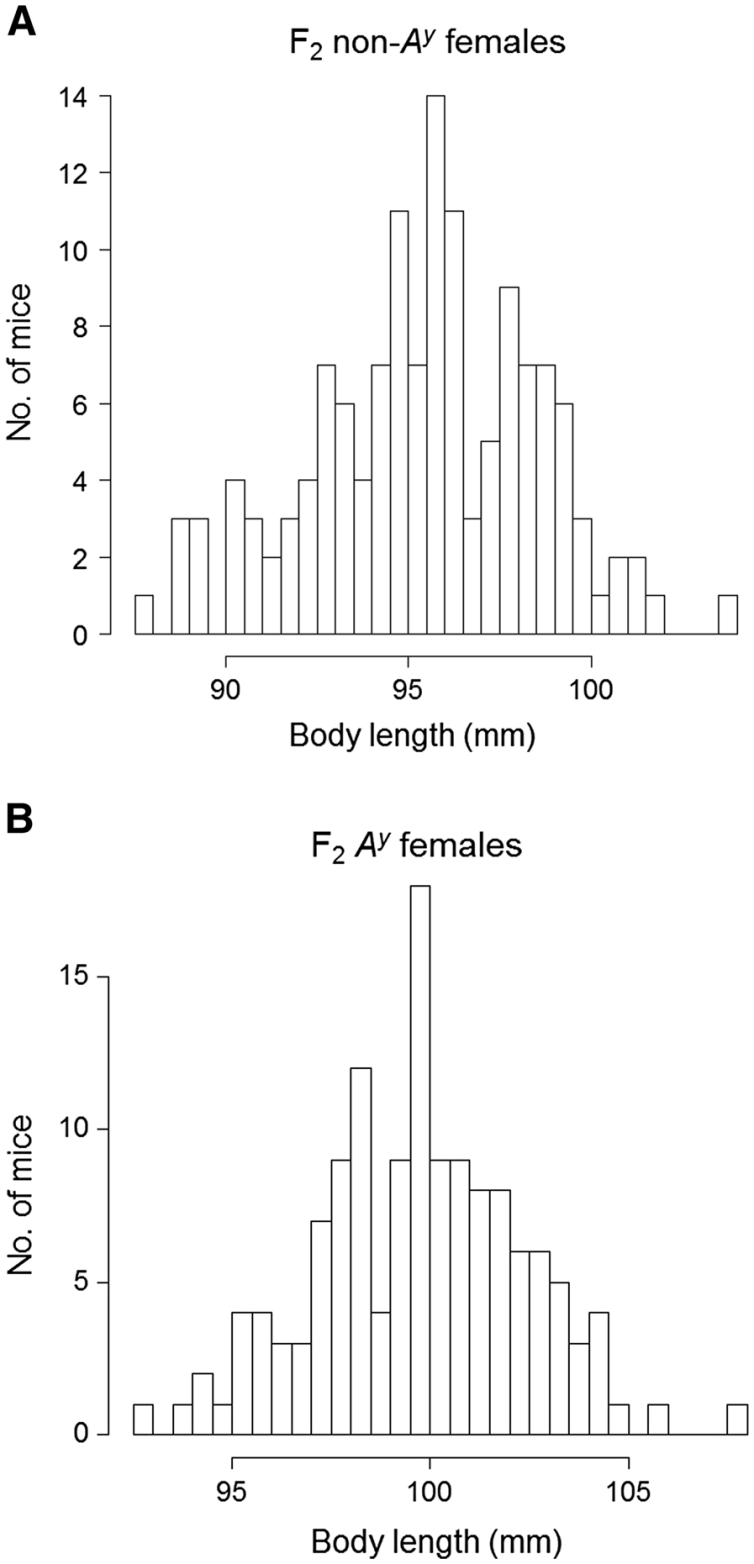
**Histograms showing the distributions of body length (mm) in F**_**2 **_**non-*A***^***y ***^**(A) and F**_**2 **_***A***^***y ***^**(B) mice**

**Figure 2:**
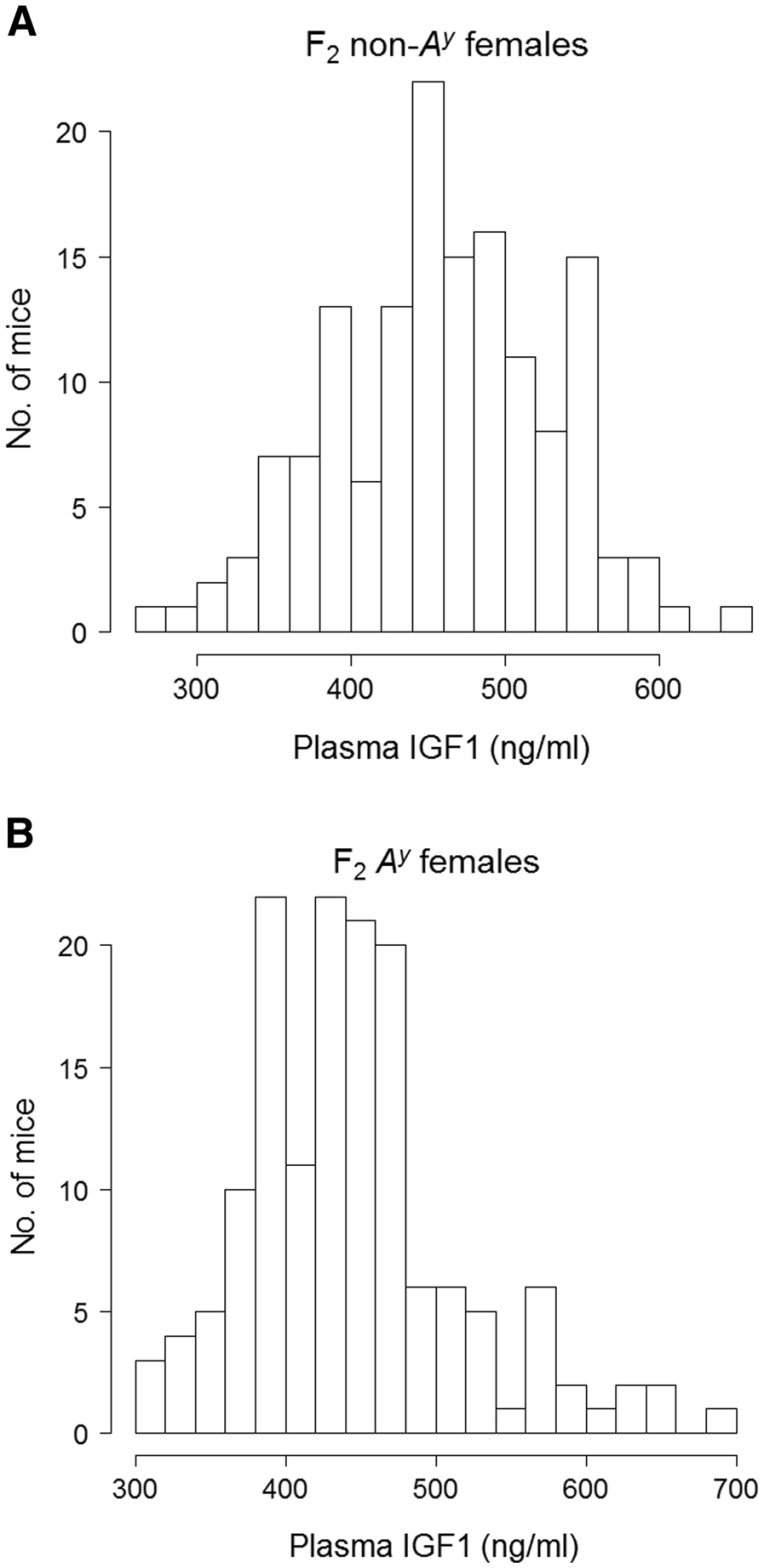
**Histograms showing the distributions of plasma IGF1 levels (ng/ml) in F**_**2 **_**non-*A***^***y ***^**(A) and F**_**2 **_***A***^***y ***^**(B) mice.**

Histograms showing the distribution of body length in F_2_ females are shown in Figure 1 [(A) F_2_ non-*A*^*y*^ females, n = 137, (B) F_2_*A*^*y*^ females, n = 139]. Mean ± S.E. body length was significantly larger in F_2_*A*^*y*^ females (99.73 ± 0.22 mm) than F_2_ non-*A*^*y*^ females (95.42 ± 0.27 mm) (P < 5.2 × 10^–28^). In combined F_2_ females (F_2_ non-*A*^*y*^ plus F_2_*A*^*y*^), body length was normally distributed. Histograms showing the distribution of IGF1 levels in F_2_ females are shown in Figure 2 [(A) F_2_ non-*A*^*y*^, n = 148, (B) F_2_*A*^*y*^ females, n = 150]. Mean ± S.E. IGF1 levels did not significantly differ between F_2_ non-*A*^*y*^ (459.5 ± 5.8 ng/dl) and F_2_*A*^*y*^ (445.6 ± 5.8 ng/dl) (P > 0.09) females. In combined F_2_ females, IGF1 levels were not normally distributed; therefore, IGF1 levels were normalized by Box-Cox transformation. Compared to a relatively high correlation between body length and body weight, correlations between body weight and IGF1 levels and between body length and IGF1 levels were modest albeit all correlations were statistically significant in both the F_2_ non-*A*^*y*^ and F_2_*A*^*y*^ females (Table 2).

**Table 2:** **Pearson coefficient of correlation in F**_**2 **_**female mice**

	**F**_**2 **_**non-*A***^***y ***^**mice**			**F**_**2 **_***A***^***y ***^**mice**	
	**Body length**	**IGF1 levels**		**Body length**	**IGF1 levels**
Body weight	0.7506 (P < 0.0001)	0.2004 (P < 0.02)	Body weight	0.6413 (P < 0.0001)	0.2708 (P < 0.002)
Body length		0.2576 (P < 0.003)	Body length		0.3077 (P < 0.0003)

**Figure 3:**
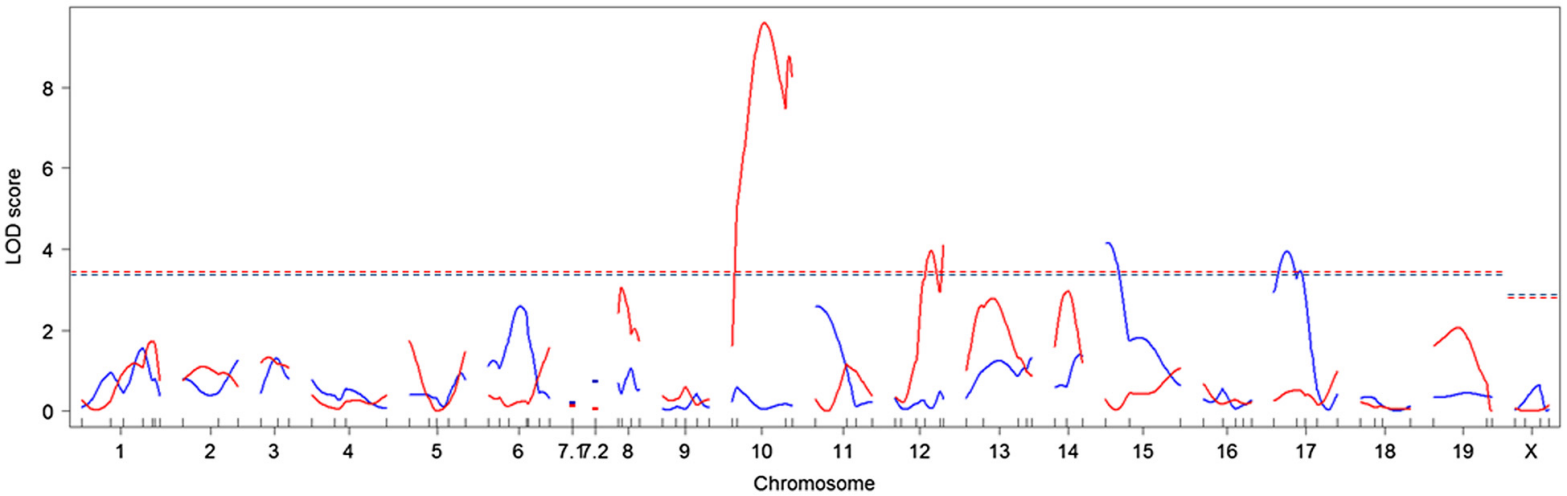
**LOD score plots for body length and IGF1 levels by single QTL scan with the agouti locus genotype as an additive covariate.** The *x*-axis shows the chromosome numbers and the *y*-axis shows the LOD scores at these locations. Blue lines indicate the LOD scores for body length, and red lines indicate the LOD scores for IGF1 levels. Horizontal dashed lines (color-coded by each trait) indicate significant threshold LOD scores determined by 1,000 permutations.

**Figure 4:**
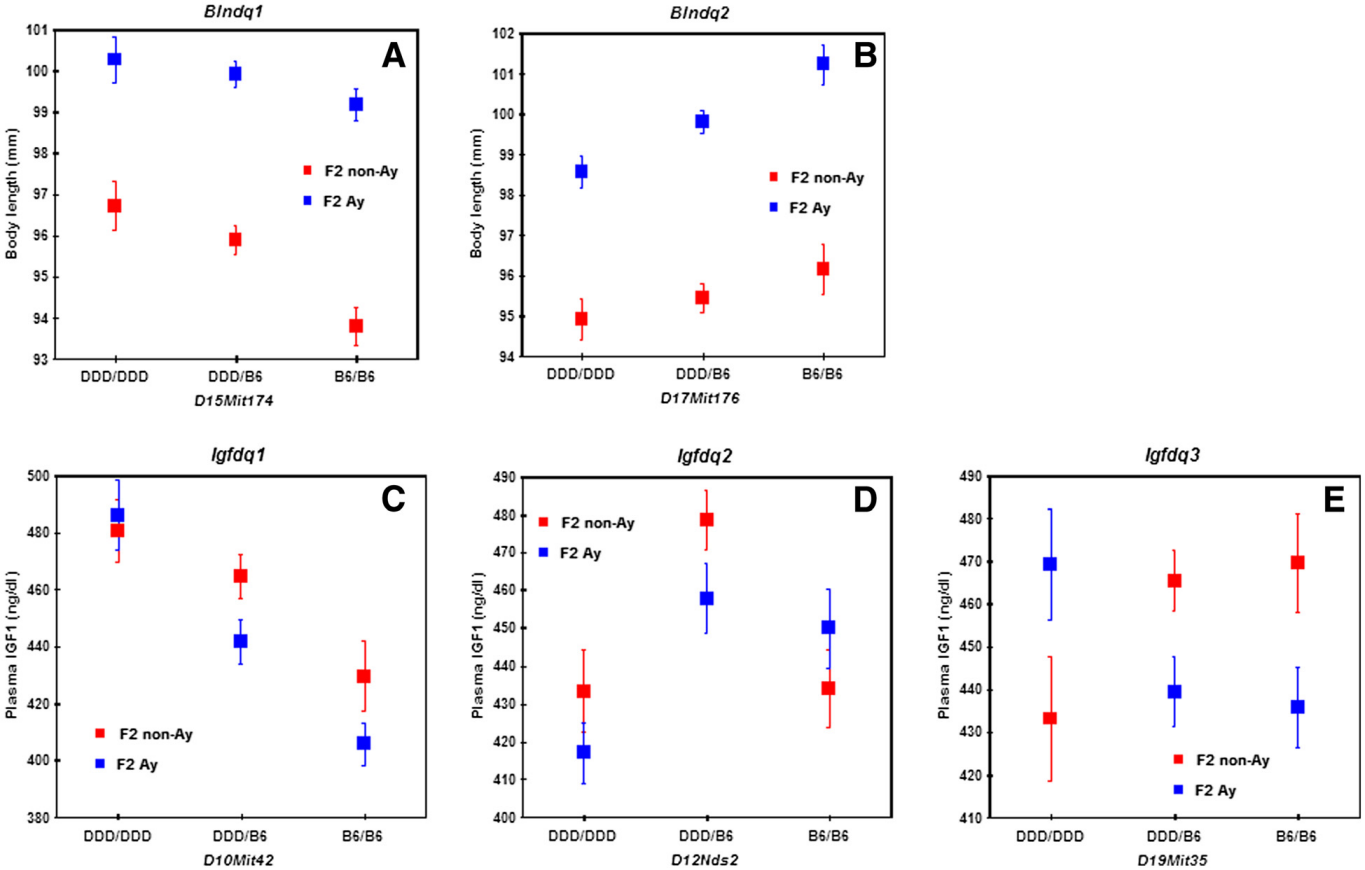
**Allelic contributions to QTLs for body length on (A) chromosomes 15 and (B) 17, and those to QTLs for IGF1 levels on (C) chromosomes 10, (D) 12, and (E) 19.** Homozygous DDD alleles are represented by DDD/DDD, homozygous B6 alleles by B6/B6, and heterozygous alleles by DDD/B6. The *y*-axes show the mean trait values and the error bars show SE.

Because the *A*^*y*^ allele had a large phenotypic effect on IGF1 levels, the agouti locus genotype (non-*A*^*y*^ or *A*^*y*^) was included as an additive covariate in the following QTL mapping analyses. For body length, two significant QTLs were identified on chromosomes 15 and 17, and two suggestive QTLs were identified on chromosomes 6 and 11 (Table 3 and Figure 3). We assigned the gene symbols *Blndq1* (body length in DDD QTL no. 1) and *Blndq2* to the significant QTLs. The DDD allele was associated with increased body length at the *Blndq1*(Figure 4A), whereas the DDD allele was associated with decreased body length at the *blndq2* (Figure 4B). There were no significant pair-wise interactions.

**Table 3:** **QTLs identified by single QTL scans with the *agouti *locus as an additive covariate**

**Trait**	**Chromosome**	**Location (cM) **^**a**^	**95% CI (cM) **^**b**^	**Max LOD **^**c**^	**Nearest marker**	**High allele **^**d**^	**Name **^**e**^
Body length	6	35	0–51	2.58	*D6Mit39*	B6	
	11	2	0–35	2.58	*D11Mit236*	DDD	
	15	3	0–20	4.16 *	*D15Mit174*	DDD	*Blndq1*
	17	14	0–37	3.94 *	*D17Mit176*	B6	*Blndq2*
IGF1 levels	8	4	0–23	3.07	*D8Mit191*	B6	
	10	36	21–66	9.60 *	*D10Mit42*	DDD	*Igfdq1*
	12	54	27–54	4.11 *	*D12Nds2*	Het	*Igfdq2*
	13	28	2–60	2.78	*D13Mit64*	DDD	
	14	15	0–29	2.96	*D14Mit193*	B6	
	19	27	0–60	2.06	*D19Mit32*	na	

^a^ Location indicates a map position showing a peak LOD score in cM.

^b^ 95% CI is defined by a 1.5-LOD support interval.

^c^ Maximum LOD score for QTL. Significant QTLs are indicated by * (suggestive QTLs are presented without asterisk).

^d^ Allele associated with higher trait values. Het, heterozygous genotype is associated with higher trait values. na, not applicable because this QTL interacts with the agouti.

^e^ Assignment of QTL name is limited to significant QTLs.

Because the difference between the LOD score with the agouti locus genotype as an interactive covariate and the LOD score with the agouti locus genotype as an additive covariate concerns the test of the QTL × agouti locus genotype interaction, this was performed. However, there were no significant QTLs that interacted with the *A*^*y*^ allele.

For IGF1 levels, two significant QTLs were identified on chromosomes 10 and 12, and four suggestive QTLs were identified on chromosomes 8, 13, 14, and 19 (Table 3 and Figure 3). We assigned the gene symbols *Igfdq1* (IGF1 levels in DDD QTL no. 1) and *Igfdq2* to the significant QTLs. The DDD allele was associated with increased IGF1 levels at the *Igfdq1* (Figure 4C), whereas the heterozygous genotype was associated with increased IGF1 levels at the *Igfdq2* (Figure 4D). There were no significant pair-wise interactions.

**Figure 5:**
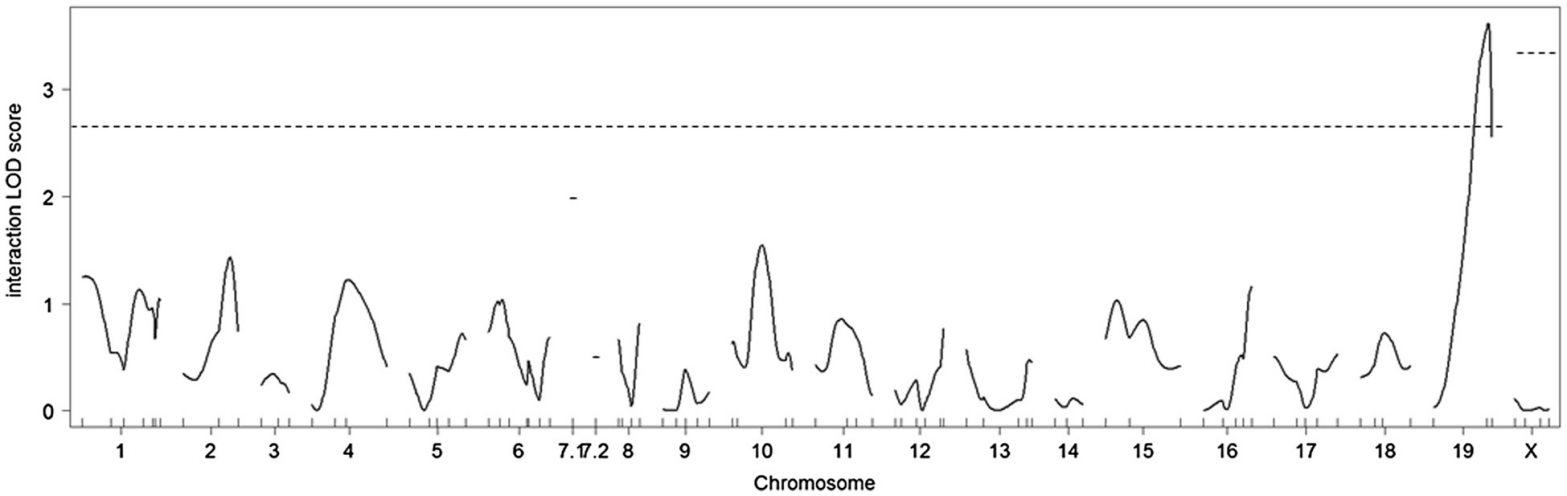
**Interaction LOD score plots for the IGF1 levels.** A horizontal dashed line indicates significant threshold LOD scores determined by 1,000 permutations. Solid lines denote the LOD scores when the agouti genotypes (non-*A*^*y*^ or *A*^*y*^) were included as covariates. Horizontal dashed lines indicate significant threshold LOD scores determined by 1,000 permutations.

When the QTL × agouti locus genotype interaction was tested, one significant interacting QTL was identified on chromosome 19 (Table 4 and Figure 5). We assigned the gene symbol *Igfdq3* to this locus. Indeed, a direction of allele effect of this QTL differed between F_2_ non-*A*^*y*^ and F_2_*A*^*y*^ females (Figure 4E). Thus, *Igfdq3* interacted with the *A*^*y*^ allele.

**Table 4:** **Summary of single QTL scans for IGF1 levels in F**_**2 **_**female mice using the *agouti *locus genotype as a covariate**

**Chromosome**	**LOD scores [peak position (cM), name]**		
	***agouti *as an additive covariate (LOD**_**a**_**) **^**a**^	***agouti *as an interactive covariate (LOD**_**f**_**) **^**b**^	**LOD**_**i**_**(LOD**_**f**_**-LOD**_**a**_**) **^**c**^
10	9.60 (36, *Igfdq1*)	11.11 (35, *Igfdq1*)	
12	4.11 (54, *Igfdq2*)	4.88 (54, *Igfdq2*)	
19			3.62 (61, *Igfdq3*)

Only significant QTLs are listed.

^a^ Significant threshold LOD scores are 3.29 for autosomes and 2.74 for X chromosome.

^b^ Significant threshold LOD scores are 4.39 for autosomes and 3.49 for X chromosome.

^c^ LOD_i_ is the difference between the LOD score with *agouti* as an interactive covariate (LOD_f_) and the LOD score with *agouti* as an additive covariate (LOD_a_). It concerns the test of the QTL × *agouti* interaction. Significant threshold LOD scores are 2.50 for autosomes and 3.06 for X chromosome.

## Discussion

*Blndq1* (chromosome 15) and *Blndq2* (chromosome 17) are major determinants of body length. These QTLs did not interact with the *A*^*y*^ allele, and the allele effect of these QTLs was in the same direction in F_2_ non-*A*^*y*^ and F_2_*A*^*y*^ mice. When the agouti locus genotype was included as a covariate in the analysis, no significant QTL × covariate interactions were identified. When the same analysis was applied to the body weight data, which was previously analyzed, no significant QTL × covariate interactions were identified. Thus, although DDD-*A*^*y*^ females were heavier and longer than B6-*A*^*y*^ mice, we could not identify any QTLs that interacted with the *A*^*y*^ allele. The effect of the *A*^*y*^ allele on size is probably rather complex in its physiologic mechanism of action. Indeed, we previously observed that the *A*^*y*^ allele decreased the size of the mandibular bone and the testis weight significantly [13,22].

In agreement with the results by Reed et al. [23], in which the correlation coefficient between body weight and body length in a mouse F_2_ intercross was 0.67, the correlation coefficient between weight and body length was similarly high in F_2_ non-*A*^*y*^ and F_2_*A*^*y*^ mice in this study. *Blndq2* colocalized with *Bwdq3*, which was identified for body weight in a previous study using the same F_2_ intercross [14]. At both the QTLs, the DDD allele was associated with decreased trait values, which suggests that these QTLs may be allelic and play a role in determining overall body size. Also, *Blndq1* colocalized with a suggestive QTL for body weight. A suggestive QTL for body length identified on chromosome 6 overlapped with a significant QTL for body weight, *Bwdq2*. Thus, there were some overlaps between QTLs for body length and body weight.

Although there are only a limited number of studies on body length as compared with studies on body weight, body length QTLs have been reported by others. Among the results of such studies, QTLs for body length have been mapped to chromosome 2 several times. Reed et al. [23] identified significant QTLs on chromosome 2 (*Bdln3*), near the agouti locus, in an F_2_ intercross between 129P3/J and C57BL/6ByJ. Farber and Medrano [24] identified a significant QTL (*Bdlnq7*) on distal chromosome 2 in an F_2_ intercross between the CAST/EiJ and B6-*hg*/*hg* strains. Chiu et al. [25] presented evidence that a significant QTL was present on distal chromosome 2 using subcongenic strains for obesity. Masinde et al. [19] identified *Lgth1* and *Lgth2* on chromosome 2 in an F_2_ intercross between the MRL/MPJ and SJL/J strains. Apparently, the agouti locus is one of the plausible candidate genes for these QTLs.

Body length QTLs were also reported for other chromosomes. Reed et al. [23] identified *Bdln6*, which was significant only for males, on chromosome 4 in the same F_2_ intercross described previously. Masinde et al. [19,20] identified *Lgth3* on chromosome 4, *Lgth4* and *Lgth5* on chromosome 9, *Lgth6* on chromosome 11, *Lgth7* and *Lgth8* on chromosome 13, and *Lgth9* on chromosome 17 in the same F_2_ intercross described previously. The 95% CI for *Lgth6* overlapped with that for suggestive QTL on chromosome 11. Similarly, *Lgth9* overlaps with *Blndq2*, which suggests that these loci are allelic.

It is important to note that more than a few of these QTLs for body length have candidate genes that were contained in growth hormone/insulin-like growth factor axis. Indeed, the 95% CI for *Blndq1* on the proximal part of chromosome 15 contains growth hormone receptor (*Ghr*) locus and the 95% CI for *Blndq2* on proximal chromosome 17 contains insulin-like growth factor 2 receptor (*Igf2r*) as candidate genes. Therefore, we next performed QTL analysis on IGF1 levels.

Surprisingly, there was no overlap between QTLs for body length and IGF1 levels. This suggests that genetic variations affecting IGF1 levels did not have substantial effects on body length. *Igfdq1* (chromosome 10) and *Igfdq2* (chromosome 12) are primary determinants of IGF1 levels. *Igfdq1* and *Igfdq2* did not interact with the *A*^*y*^ allele, and the allele effect of these QTLs was in the same direction in F_2_ non-*A*^*y*^ and F_2_*A*^*y*^ mice. In contrast, *Igfdq3* interacted with the *A*^*y*^ allele. The 95% CI for *Igfdq3* contains Kazal-type serine peptidase inhibitor domain 1 (*Kazald1*), which is also known as *Igfbp-rp10*, as a candidate gene. *Kazald1* is shown to promote proliferation of osteoblasts during bone formation and bone regeneration [26].

To date, four other studies have addressed blood (plasma or serum) IGF1 levels in mouse intercrosses [27-30]. Brockmann et al. [27] identified two significant QTLs on chromosomes 10 and 18 in an F_2_ intercross between Du6i and DBA/2 mice. They reported that chromosomal regions harboring these QTLs did not show any linkage to body, muscle, or fat weight. Rosen et al. [28] identified three significant QTLs on chromosomes 6, 10, and 15 in an F_2_ intercross between C3H/HeJ and B6. Harper et al. [29] identified five significant QTLs on chromosomes 1, 3, 8, 10, and 17 in genetically heterogeneous mice. Leduc et al. [30] identified four significant QTLs on chromosomes 9, 10, 15, and 17 in F_2_ mice between MRL/MpJ and SM/J. Thus, *Igfdq2* (chromosome 12) and *Igfdq3* (chromosome 19), identified in this study, are considered to be novel QTLs for blood IGF1 levels. In particular, *Igfdq3* was shown to interact with the *A*^*y*^ allele. Most importantly, all five studies, including the present study, identified a significant QTL on chromosome 10 at a position containing the *Igf1* locus, and it was considered that the *Igf1* itself was a plausible candidate gene for the QTL. To determine whether or not *Igf1* is responsible for *Igfdq2*, further studies, including sequence and expression analyses, will be required [28,30,31].

Finally, we could not analyze the distal portion of chromosome 2 (surrounding the agouti locus) and the mid-part of chromosome 7 (surrounding tyrosinase locus) because these chromosomal regions in DDD-*A*^*y*^ strain were derived from B6-*A*^*y*^ strain. In particular, the allele at the distal chromosome 2 was biased toward the B6 allele in F_2_ non-*A*^*y*^ mice, whereas the allele was biased toward the DDD allele in F_2_*A*^*y*^ mice. Also, we could not sufficiently analyze the entire part of X chromosome due to the cross direction of parental strains. Therefore, we cannot deny a possibility that there are additional QTLs in these chromosomal regions. Most importantly, because the present study was conducted in females, the QTL effect may not necessarily be confirmed in males. We are currently establishing QTL congenic strains to confirm the presence and effect of the QTLs in both sexes.

## Conclusion

In summary, QTLs for body length and IGF1 levels contained candidate genes that were components of the growth hormone/insulin-like growth factor axis. However, there was no overlap between QTLs for each trait. Contrary to our expectations, a QTL that interacted with the *A*^*y*^ allele was identified not for body length but for IGF1 levels. Body length and IGF1 levels were thus controlled by different sets of genes.

## Methods

### Mice

The inbred mouse DDD strain (agouti locus genotype, *A*/*A*) and the congenic mouse DDD-*A*^*y*^ strain (*A*^*y*^/*A*) were maintained at the National Institute of Agrobiological Sciences (NIAS). The inbred mouse C57BL/6 J strain (hereafter designated B6, *a*/*a*) was purchased from Clea Japan (Clea Japan Inc., Tokyo). The congenic mouse B6-*A*^*y*^ strain (*A*^*y*^/*a*) was purchased from The Jackson Laboratory (Bar Harbor, ME, USA). The DDD-*A*^*y*^ strain was established by introgressing the *A*^*y*^ allele from the B6-*A*^*y*^ strain in the DDD strain by backcrossing for 12 generations [13]. Hereafter, both DDD-*A*^*y*^ and B6-*A*^*y*^ are referred to as “*A*^*y*^ mice.” Similarly, their control littermates, DDD and B6, are referred to as “non-*A*^*y*^ mice.”

DDD-*A*^*y*^ males were crossed with B6 females to produce the F_1_ generation, and F_1_*A*^*y*^ (*A*^*y*^/*a*) mice were intercrossed with F_1_ non-*A*^*y*^ (*A*/*a*) mice to produce the F_2_ generation. F_2_ females were weaned at 4 weeks. The mice were housed in groups of 4–5 for 16 weeks.

All mice were maintained in a specific pathogen-free facility with a regular light–dark cycle (12 h light and 12 h dark) and controlled temperature (23 ± 1°C) and humidity (50%). Food (CRF-1; Oriental Yeast Co Ltd., Tokyo, Japan) and water were freely available throughout the experimental period. All animal experiments were performed in accordance with the guidelines of the Institutional Animal Care and Use Committee of NIAS.

### Phenotyping

At the age of 16 weeks, the body weight of the mice, fasted for 4 h, was determined using an electric balance to the nearest 0.01 g. The mice were euthanized with an overdose of ether. Whole blood was drawn from the heart into a plastic tube using heparin as an anticoagulant. Sample tubes were centrifuged at 7,000 rpm for 5 min at −4°C to separate the plasma. The plasma samples were maintained at −80°C until use. The IGF1 concentration was determined by ELISA (R&;D Systems, Inc., Minneapolis, MN55413, USA). The anal–nasal length of each mouse was measured by a pair of digital calipers to the nearest 0.01 mm just after the blood collection to avoid rigor mortis. Body length is defined as the anal–nasal length in this study.

Normality of the distribution of trait data for combined F_2_ females (F_2_ non-*A*^*y*^ plus F_2_*A*^*y*^, n = 298) was tested using the Shapiro–Wilk W test (JMP 8.0.2, SAS Institute Japan, Tokyo, Japan). If the trait values did not follow a normal distribution, they were appropriately normalized using the Box–Cox transformation.

### Genotyping and QTL analysis

Genomic DNA isolation and genotyping of microsatellite markers were performed according to the procedure described in our previous study [14]. Microsatellite markers used in this study were listed in the Table 5 with their map position (cM) calculated using combined F_2_ females.

**Table 5:** Genetic markers and their map positions used in this study

**Marker **^**a**^	**Map position **^**b**^	**Marker**	**Map position**	**Marker**	**Map position**	**Marker**	**Map position**
Chromosome 1		Chromosome 6		Chromosome 11		Chromosome 16	
*D1Mit231*	0	*D6Mit116*	0	*D11Mit236*	0	*D16Mit131*	0
*D1Mit303*	31.6	*D6Mit224*	12.2	*D11Mit36*	34.6	*D16Mit57*	21.8
*D1Mit10*	46.1	*D6Mit188*	22.6	*D11Mit124*	44.8	*D16Mit136*	36.1
*D1Mit102*	67.2	*D6Mit39*	41.7	*D11Mit61*	62.6	*D16Mit139*	44.3
*D1Mit16*	77.7	*D6Mit108*	44.0			*D16Mit49*	53.0
*Apoa2*	80.2	*D6Mit256*	55.7	Chromosome 12			
*D1Mit291*	86.1	*D6Mit259*	67.2	*D12Mit136*	0	Chromosome 17	
				*D12Mit172*	6.6	*D17Mit164*	0
Chromosome 2		Chromosome 7.1		*D12Mit156*	23.4	*D17Mit176*	25.1
*D2Mit312*	0	*D7Mit250*	0	*D12Mit259*	32.7	*D17Mit139*	33.9
*D2Mit296*	38.9			*D12Mit141*	49.3	*D17Mit93*	47.5
*D2Mit92*	60.7	Chromosome 7.2		*D12Nds2*	53.6	*D17Mit123*	69.5
		*D7Mit362*	0				
Chromosome 3				Chromosome 13		Chromosome 18	
*D3Mit203*	0	Chromosome 8		*D13Mit207*	0	*D18Mit21*	0
*D3Mit25*	18.5	*D8Mit191*	0	*D13Mit64*	18.8	*D18Mit149*	15.7
*D3Mit212*	30.3	*D8Mit205*	3.6	*D13Mit110*	56.5	*D18Mit152*	23.9
		*D8Mit249*	14.5	*D13Mit213*	66.2	*D18Mit25*	54.8
Chromosome 4		*D8Mit183*	23.2	*D13Mit171*	71.3		
*D4Mit1*	0					Chromosome 19	
*D4Mit178*	26.0	Chromosome 9		Chromosome 14		*D19Mit32*	0
*D4Mit166*	37.3	*D9Mit59*	0	*D14Mit64*	0	*D19Mit91*	59.0
*D4Mit234*	82.3	*D9Mit191*	14.9	*D14Mit193*	13.3	*D19Mit35*	65.0
		*D9Mit207*	25.4	*D14Mit165*	30.7		
Chromosome 5		*D9Mit198*	38.2			Chromosome X	
*D5Mit267*	0	*D9Mit212*	51.3	Chromosome 15		*DXMit166*	0
*D5Mit113*	21.6			*D15Mit174*	0	*DXMit119*	11.2
*D5Mit239*	30.2	Chromosome 10		*D15Mit184*	26.3	*DXMit64*	27.2
*D5Mit161*	43.0	*D10Mit188*	0	*D15Mit193*	82.2	*DXMit38*	36.7
*D5Mit221*	61.6	*D10Mit183*	5.6				
		*D10Mit42*	59.1				
		*D10Mit95*	66.3				

^a^*Apoa2* (apolipoprotein A-II) was genotyped with a PCR-RFLP method according to the Suto et al. [32] procedure.

^b^ Map positions (cM) were based on a linkage map calculated using combined F_2_ females (n = 298).

It should be noted that chromosome 7 is divided into two parts. Due to the introgression of the *Tyr* locus from the B6 strain, a mid-part of the DDD genome on chromosome 7 is replaced by a B6 genome in DDD-*A*^*y*^ mice. In this study, a region proximal to the B6 region was defined as “chromosome 7.1 (*D7Mit250*).” whereas a region distal to the B6 region was defined as “chromosome 7.2 (*D7Mit362*).”

Of a total of 298 F_2_ females, 148 were F_2_ non-*A*^*y*^ and 150 were F_2_*A*^*y*^ mice. QTL analysis was performed using R/qtl [33,34]. Data for F_2_ non-*A*^*y*^ and F_2_*A*^*y*^ mice were combined and analyzed using the agouti locus genotype (i.e., *A*^*y*^ or non-*A*^*y*^) as a covariate. Threshold logarithm of odds (LOD) scores for suggestive (P < 0.63) and significant (P < 0.05) linkages were determined by performing 1,000 permutations for each trait [35]. For significant QTLs, a 95% confidence interval (CI) was defined by a decline of 1.5 LOD. After these single QTL scans, pair-wise evaluations for potential interactions between loci were made. At this stage, threshold LOD scores were based strictly on those recommended by Broman in “A Brief Tour of R/qtl” ( http://www.rqtl.org).

### Other statistics

Statistical analysis for the two groups was performed using Student’s or Welch’s t-test, while that for more than two groups was performed using Tukey-Kramer HSD test (JMP 8, SAS Institute Inc., Cary, NC 27513, USA). Strength of association between body weight and body length was evaluated by using the Pearson product moment correlation coefficient. P < 0.05 was considered to be statistically significant.

## Competing interests

The author declares that he has no competing interests.
